# Diff-MomentFormer: Generative Diffusion-Augmented Transformer for End-to-End Joint Moment Estimation

**DOI:** 10.3390/s26102944

**Published:** 2026-05-08

**Authors:** Chengyu Qiao, Eryun Liu, Jingwei Ren, Long He

**Affiliations:** 1 The College of Information Science and Electronic Engineering, Zhejiang University, Hangzhou 310027, China; 3140104437@zju.edu.cn; 2 Hangzhou Zhiyuan Research Institute, Hangzhou 310012, China; renjingwei@zy-cs.com.cn

**Keywords:** joint moment estimation, classifier-free conditional diffusion, Transformer, multimodal sensor fusion, lower-limb exoskeleton

## Abstract

Accurate estimation of human joint moments from multimodal sensor signals is essential for lower-limb exoskeleton control. Recent studies have addressed this problem in an end-to-end manner, but remain limited by insufficient long-range temporal modeling, limited training data, and class imbalance. To address these issues, we propose Diff-MomentFormer, a generative diffusion-augmented Transformer framework for end-to-end joint moment estimation from multimodal wearable sensor signals. The framework integrates a classifier-free conditional diffusion model for activity-conditioned synthetic data generation, together with a Transformer-based regression network for modeling long-range temporal dependencies and cross-modal interactions. Through the combination of controllable data augmentation and global temporal modeling, Diff-MomentFormer can learn more robust multimodal representations for accurate and stable joint moment estimation. Extensive experiments on a public lower-limb biomechanics dataset show that the proposed method consistently improves hip and knee joint moment estimation performance across different activity categories, while ablation studies further confirm the effectiveness of the proposed framework.

## 1. Introduction

With the rapid development of robotics, wearable sensing technologies, and intelligent control algorithms, lower-limb exoskeletons have shown great potential in human assistance [1,2,3,4,5], rehabilitation [6,7], and physical augmentation [8,9]. In industrial and physically demanding scenarios, they can provide assistance to reduce muscular load, improve operational efficiency, and lower the risk of musculoskeletal injury [3,10,11]. In rehabilitation and mobility support, they can facilitate motor recovery and help maintain long-term functional independence through precise biomechanical assistance [6,12]. These application demands highlight the importance of effective lower-limb exoskeleton control, particularly for enabling accurate, adaptive, and natural human–robot interaction.

As a core component of lower-limb exoskeleton systems, control strategies have attracted extensive research attention [13,14]. Conventional controllers typically follow a hierarchical architecture [15], in which locomotion modes are first recognized at the high level [16,17,18,19,20,21], assistive torques are then generated at the mid-level using predefined rules or state-machine logic [5,22,23], and the resulting commands are finally executed by the low-level actuator controller. Although such pipelines have shown satisfactory performance in structured laboratory settings, they rely heavily on handcrafted rules and careful parameter tuning. As a result, their adaptability and generalization are often limited when facing diverse movement patterns, transition phases, and inter-subject variability. Recent studies have therefore increasingly explored data-driven approaches that estimate joint moments from multimodal sensor signals in an end-to-end manner [24,25,26,27,28,29,30], providing a promising route toward more continuous and subject-adaptive exoskeleton control.

End-to-end joint moment estimation aims to directly predict lower-limb joint moments from multimodal time-series signals collected by exoskeleton-embedded sensors, such as inertial measurement units (IMUs), joint encoders, and plantar-pressure sensors [29,30]. During training, the models are supervised by ground-truth joint moments computed from inverse dynamics using motion-capture data and ground reaction force (GRF) measurements. Compared with conventional pipelines that depend on explicit locomotion-mode recognition or phase-based parameterization, this paradigm enables continuous and task-agnostic modeling of user biomechanics and provides a more direct basis for assistive torque generation. Existing studies have shown that temporal convolutional network (TCN)-based estimators can achieve accurate hip and knee joint moment prediction across diverse movement conditions and can be integrated into closed-loop exoskeleton control, demonstrating the practical promise of this direction.

Despite this progress, two key challenges remain. First, current methods do not fully exploit the long-range temporal dependencies and cross-modal interactions inherent in multimodal exoskeleton sensor signals. Most existing methods rely on convolutional neural networks (CNNs) as the backbone. Although CNNs are effective at extracting local temporal patterns, they are less suited to modeling long-range dependencies and complex coupling relationships across sensor modalities. Second, the availability of high-quality labeled data remains limited. Ground-truth joint moments must typically be obtained through synchronized motion-capture and force measurements, which makes data collection costly and restricts the scale of usable training data. In addition, publicly available datasets often provide limited coverage of rare or highly dynamic activities, leading to class imbalance and reduced generalization on underrepresented motion types. Therefore, improving end-to-end joint moment estimation in real-world scenarios requires both stronger temporal and multimodal modeling at the network level and more effective data augmentation at the data level.

Recent advances in Transformers and diffusion models provide a promising foundation for addressing these challenges. Transformer architectures [31] have demonstrated strong sequence modeling capability across a wide range of domains, largely because the self-attention mechanism enables global dependency modeling between any pair of positions in an input sequence. This property makes Transformers particularly suitable for capturing long-range temporal correlations and cross-modal interactions in multimodal exoskeleton sensor data. At the same time, diffusion models [32] have emerged as a powerful class of generative models for learning complex data distributions and synthesizing high-quality samples from random noise. In particular, classifier-free conditional guidance [33] enables controllable generation without requiring an auxiliary classifier, making diffusion models especially attractive for class-conditioned data augmentation and distribution balancing. Together, these advances suggest a natural way to improve end-to-end joint moment estimation by strengthening temporal modeling and enriching training data simultaneously.

**Figure 1:**
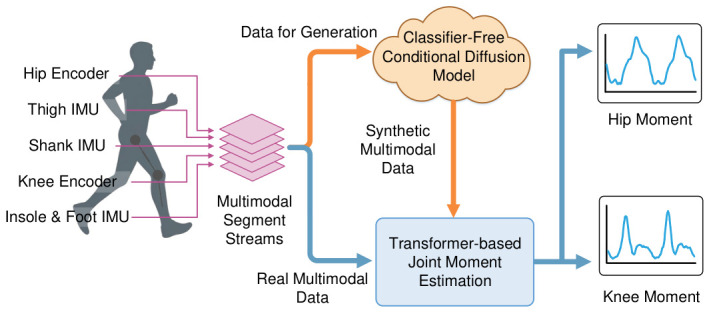
Basic pipeline of Diff-MomentFormer. Multimodal sensor data are first used to train a classifier-free conditional diffusion model for synthetic data generation, and the resulting synthetic data are then fused with real data for Transformer-based joint moment estimation.

To address the above challenges, we propose Diff-MomentFormer, a generative diffusion-augmented Transformer framework for end-to-end joint moment estimation. Figure 1 illustrates the overall pipeline of the proposed framework. Specifically, we first train a classifier-free conditional diffusion model to learn class-conditioned multimodal temporal distributions and generate category-specific synthetic samples for data augmentation. The generated samples are then fused with real measurements to train a Transformer-based joint moment estimation network, which is designed to better capture long-range temporal dependencies and cross-modal interactions in multimodal sensor signals. Under this design, the diffusion module alleviates data scarcity and class imbalance, while the Transformer module enhances temporal modeling and multimodal feature fusion. Together, these two components enable more accurate and stable joint moment estimation, especially under complex movements and task transitions.

The main contributions of this work are summarized as follows:

1. We introduce conditional diffusion-based augmentation into the joint moment estimation pipeline, enabling class-conditioned and controllable generation of synthetic multimodal temporal data to alleviate data scarcity and class imbalance.
2. We develop a Transformer-based end-to-end joint moment estimation network that more effectively captures long-range temporal dependencies and cross-modal feature interactions, thereby improving estimation stability and accuracy.
3. We validate the proposed framework through extensive experiments on a public lower-limb biomechanics dataset, demonstrating consistent performance improvements over counterpart models without data augmentation or architectural improvements.

## 2. Related Work

### 2.1. Joint Moment Estimation

In recent years, increasing attention has been devoted to data-driven joint moment estimation and its integration into exoskeleton control frameworks. Compared with conventional mode-dependent control strategies, these approaches reduce reliance on explicit locomotion classification and manual parameter tuning, thereby improving adaptability and usability in real-world scenarios. Gasparri et al. [25] designed an ankle exoskeleton controller by fitting the relationship between foot-force measurements and ankle moments during level walking, enabling adaptive and real-time torque modulation. Subsequent studies extended this idea to more complex tasks, including incline and decline walking, stair ascent and descent, and mixed terrain locomotion, showing promising energetic benefits and practical applicability for both able-bodied users and clinical populations [34,35,36]. In parallel, energy-shaping methods provided an alternative route for multi-joint assistance based on joint motion, offering a more unified control strategy across different lower-limb joints [24,37].

With the development of wearable sensing and deep learning, end-to-end estimation of biomechanical quantities from sensor measurements has become increasingly feasible. Dorschky et al. [28] demonstrated that CNNs could be used to estimate joint angles, joint moments, and ground reaction forces from inertial sensing data collected from simulated and experimental sources. Molinaro et al. [26] first introduced a temporal convolutional network (TCN)-based estimator for hip joint moments during multimodal overground ambulation. They systematically evaluated the model’s performance under diverse steady-state walking conditions. Subsequent work further investigated how temporal history affects delayed estimation and anticipation of sagittal-plane hip moments [27]. Building on these findings, they integrated TCN-based estimation with mid-level control strategies to build a unified framework for robotic hip exoskeleton assistance [29]. This framework achieved accurate moment estimation across multiple walking modes while reducing user metabolic cost. Later studies expanded the task set to a larger collection of 28 activities, including both cyclic and unstructured motions, and achieved improved task-agnostic estimation and control of multi-joint moments such as hip and knee moments [30]. To reduce the burden of collecting high-quality labeled data, deep domain adaptation has been used to transfer open-source biomechanics data to device-specific sensor domains, thereby improving model performance while reducing the demand for costly labeled measurements on the target device [38].

Overall, these studies have made substantial progress in dataset construction, in-the-loop validation, and real-world relevance. However, most existing approaches still rely on convolutional temporal backbones, which are less effective at modeling long-range dependencies and cross-modal interactions in multimodal exoskeleton signals. Moreover, under limited access to labeled data, it remains underexplored how to learn motion-category-conditioned temporal distributions and generate diverse, high-continuity multimodal data for effective augmentation and class balancing. Motivated by these limitations, the present work focuses on enhancing temporal modeling and introducing more effective generative data augmentation to develop a more generalizable and stable joint moment estimation framework for complex movements and task transitions.

### 2.2. Transformer

Transformers have emerged as a powerful backbone for sequence modeling across a wide range of deep learning tasks. Their core self-attention mechanism enables flexible and effective modeling of long-range dependencies, while also showing strong scalability as data and model sizes increase. In natural language processing, the Transformer architecture demonstrated breakthrough performance in machine translation [31] and later drove the development of large-scale pretrained language models [39,40]. In computer vision, Vision Transformer (ViT) [41] demonstrated that images can be represented as patch sequences and modeled effectively with pure Transformer architectures, while Detection Transformer (DETR) [42] reformulated object detection as a set prediction problem within an end-to-end Transformer encoder–decoder framework. In speech processing, Transformers have also been widely adopted for sequence modeling, where self-attention helps capture long-range acoustic context and can be combined with convolutional modules to jointly model local and global temporal structure [43,44]. More recently, Transformers have been incorporated into generative modeling, for example in Diffusion Transformer (DiT) [45] and Latent Diffusion Models (LDM) [46], further highlighting their flexibility and scalability. Motivated by these advances, Transformers provide a natural solution for modeling complex multimodal time-series data. In particular, their ability to capture global temporal dependencies and cross-modal interactions makes them well-suited for exoskeleton sensor signals, which exhibit strong temporal coupling and inter-sensor correlations. Therefore, in this work, we adopt a Transformer-based architecture to enhance temporal modeling and multimodal feature fusion for end-to-end joint moment estimation.

### 2.3. Diffusion Models

Diffusion models have achieved remarkable progress in generative modeling in recent years, showing strong capability in distribution learning and sample synthesis. Ho et al. [32] first established diffusion probabilistic models as a powerful framework for high-fidelity image synthesis, and subsequent developments such as the Denoising Diffusion Implicit Model (DDIM) improved sampling efficiency by introducing a non-Markovian reverse process while preserving the original training objective [47]. For conditional generation, classifier guidance was proposed to steer the reverse process using gradients from an auxiliary classifier [48]. Classifier-free guidance later provided a unified framework that jointly learns conditional and unconditional generation and enables a practical trade-off between sample quality and diversity without requiring an additional classifier [33]. Building on these developments, diffusion models have been extended to a broad range of conditional and image-to-image tasks, including text-guided synthesis [49], image restoration [50], and realistic image editing [51]. Diffusion models have also shown strong potential in one-dimensional signal generation and speech synthesis, as demonstrated by DiffWave for raw audio waveform generation [52]. However, compared with image and speech applications, diffusion-based generation of multimodal physical-world time-series signals remains relatively underexplored. In such scenarios, generated signals must not only preserve temporal continuity within each channel, but also maintain strict synchronization and coupling relationships across different sensor modalities. This makes multimodal biomechanical sensing a particularly challenging yet promising application domain for diffusion models. In this work, we investigate the use of diffusion models for multimodal temporal data generation and augmentation in exoskeleton-related sensing scenarios, with the goal of enriching the training set, alleviating class imbalance, and improving the robustness of downstream end-to-end joint moment estimation.

## 3. Method

**Figure 2:**
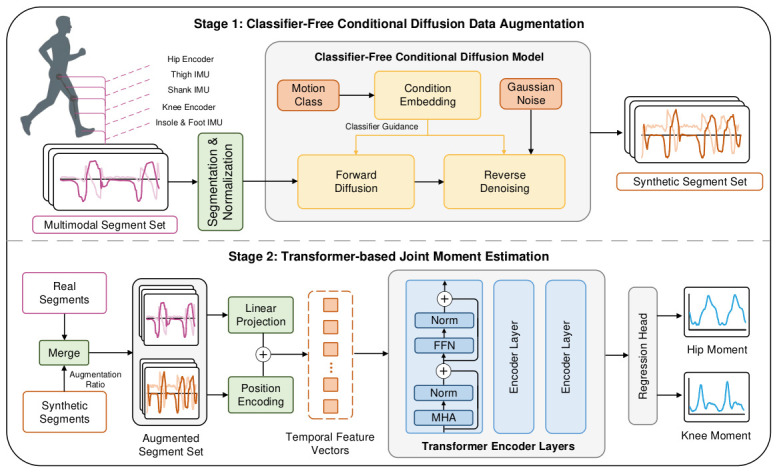
Overall architecture of Diff-MomentFormer. The framework consists of two main components: (1) a classifier-free conditional diffusion module that learns class-conditioned multimodal temporal distributions and generates synthetic segments containing both multimodal sensor signals and the corresponding joint-moment sequence for data augmentation; and (2) a Transformer-based joint moment estimation module that fuses real and synthetic segments to model long-range temporal dependencies and multimodal interactions for hip and knee joint moment estimation.

As illustrated in Figure 2, Diff-MomentFormer consists of two core modules: (i) a classifier-free conditional diffusion model for data augmentation and (ii) a Transformer-based joint moment estimation module. Given a set of variable-length multimodal motion sequences $S={\left\{s_{i}\right\}}_{i=1}^{M}$, where $s_{i}$ denotes the *i*-th raw multimodal sequence formed by the multimodal sensor signals and the corresponding ground-truth joint-moment sequence, we first preprocess the raw data and convert them into a fixed-length segment set $X={\left\{x_{i}\right\}}_{i=1}^{N}$. A classifier-free conditional diffusion model is then trained on X to learn the class-conditioned distribution of multimodal temporal segments and generate synthetic segment samples $X^{\prime }$ under motion-class conditions. In the subsequent joint moment estimation module, real and synthetic samples are fused to train a Transformer-based regression network, which captures long-range temporal dependencies and cross-modal interactions in multimodal sensor signals and outputs the corresponding joint moment predictions. The following sections provide detailed descriptions of the preprocessing, classifier-free conditional diffusion for data augmentation, Transformer-based joint moment estimation, and the corresponding loss function.

### 3.1. Multimodal Time-Series Preprocessing and Representation

Due to variations in recording duration across subjects and trials, the raw data consists of multiple variable-length sequences. To obtain a unified representation for subsequent modeling, each raw sequence is converted into a set of fixed-length temporal segments, which are further normalized along the feature dimension. This preprocessing step reduces scale differences across sensor channels and inter-subject variability, providing a consistent input space for both diffusion-based data generation and Transformer-based joint moment estimation.

Specifically, the raw input at each time step includes IMU measurements from the foot, shank, and thigh, plantar-pressure signals, and encoder readings from the hip and knee joints. These multimodal sensor signals are concatenated along the feature dimension and paired with the corresponding ground-truth joint moments to form the raw sample set, denoted as $S={\left\{s_{i}\right\}}_{i=1}^{M}$, where $s_{i}$ is the *i*-th raw multimodal motion sequence. In addition, the subject’s body weight is repeated over all time steps and concatenated with the sequence features as an auxiliary input. Each variable-length sequence is then sequentially segmented into fixed-length temporal windows of length $T_{s}$, yielding a segment set $X={\left\{x_{i}\right\}}_{i=1}^{N}$, where $x_{i}\in R^{T_{s}\times D}$ denotes the *i*-th segment containing both the multimodal sensor signals and the aligned joint-moment sequence, and *D* is the total feature dimension of the joint representation.

After segmentation, for the conditional diffusion model only, we apply dimension-wise min-max normalization to each segment. This design is adopted because the diffusion model requires inputs in the range [−1,1], where the value ranges of multimodal signals vary substantially across motion classes. Using a single global minimum and maximum for all classes would compress the dynamic range of some categories, making it difficult to preserve their characteristic temporal patterns. Therefore, for each motion class *c* and each feature dimension d∈{1,…,D}, the minimum and maximum values are computed from the training samples of class *c*, denoted as $m_{c,d}^{\min}$ and $m_{c,d}^{\max}$, respectively. For any segment $x_{i}$ belonging to class *c*, the normalized representation ${\bar{x}}_{i}$ is defined as

(1) $${\bar{x}}_{i}\left(t,d\right)=\frac{x_{i}\left(t,d\right)-m_{c,d}^{\min}}{m_{c,d}^{\max}-m_{c,d}^{\min}},\;t=1,\ldots ,T_{s}$$

After this transformation, each feature dimension is normalized to [0,1] and then linearly rescaled to [−1,1] before being used as the clean sample for the diffusion model, which facilitates stable optimization and subsequent class-conditioned temporal generation.

### 3.2. Data Augmentation via Conditional Diffusion Models

#### 3.2.1. Diffusion Preliminaries

Diffusion models learn the underlying data distribution through a forward noising process and a corresponding reverse denoising process. In the forward process, Gaussian noise is gradually added to a clean sample $x_{0}∼q\left(x_{0}\right)$ through a Markov chain of length *T*, resulting in a sequence of latent variables $x_{1},x_{2},\ldots ,x_{T}$. When *T* is sufficiently large, the terminal state $x_{T}$ approaches a standard Gaussian distribution. The forward process is defined as

(2) $$q\left(x_{t}|x_{t-1}\right)=N\left(x_{t};\sqrt{\alpha_{t}}x_{t-1},\left(1-\alpha_{t}\right)I\right)$$

where ${\left\{\alpha_{t}\right\}}_{t=1}^{T}$ denotes a predefined noise schedule. The generative objective is to learn a parameterized reverse process $p_{\theta }\left(x_{t-1}|x_{t}\right)$ that progressively removes noise and reconstructs samples from the data distribution. A common practice is to train a neural network $\epsilon_{\theta }\left(x_{t},t\right)$ to predict the noise added at diffusion step *t*. Under this parameterization, the training objective can be simplified to a mean-squared error loss:

(3) $$L=E_{x_{0},\;t,\;\epsilon }\left[∥\epsilon -\epsilon_{\theta }\left(x_{t},t\right){∥}_{2}^{2}\right]$$

where ϵ∼N(0,I) and *t* is uniformly sampled from {1,…,T}. After training, new samples can be generated by initializing the reverse process with Gaussian noise $x_{T}∼N\left(0,I\right)$ and iteratively applying the learned denoising transitions to obtain $x_{0}$.

#### 3.2.2. Conditional Diffusion Modeling

To enable controllable generation of specific motion patterns, we extend the basic diffusion formulation to a class-conditioned setting and model the conditional distribution p(x∣c), where *c* denotes the motion class label. Given an original sample $x_{0}\in R^{T_{s}\times D}$, the corresponding noisy sample at diffusion step *t* is constructed as follows:

(4) $$x_{t}=\sqrt{{\bar{\alpha }}_{t}}\;x_{0}+\sqrt{1-{\bar{\alpha }}_{t}}\;\epsilon$$

where ${\bar{\alpha }}_{t}=\prod_{s=1}^{t}\alpha_{s}$ is the cumulative noise schedule and ϵ∼N(0,I) is standard Gaussian noise.

To incorporate class information, we adopt a classifier-free conditioning strategy with a learnable class embedding layer. Let $E\in R^{K\times d}$ denote the embedding matrix, where *K* is the number of motion classes and *d* is the embedding dimension. For a class label *c*, the corresponding class embedding is defined as follows:

(5) $${\mathrm{emb}}_{\mathrm{cls}}\left(c\right)=E\left[c\right]$$

where E[c] denotes the *c*-th row of the embedding matrix. We also introduce a learnable null embedding ${\mathrm{emb}}_{\mathrm{null}}\in R^{d}$ to represent the unconditional branch. During training, a gating variable g∼Bernoulli(p) is sampled to randomly switch between the conditional and unconditional inputs, yielding the final condition embedding:

(6) $${\mathrm{emb}}_{c}=g\cdot {\mathrm{emb}}_{\mathrm{cls}}\left(c\right)+\left(1-g\right)\cdot {\mathrm{emb}}_{\mathrm{null}}$$

This design allows the model to be trained jointly under both conditional and unconditional settings, which enables classifier-free guidance during inference. To encode diffusion-step information, we adopt a one-dimensional sinusoidal positional encoding for the time embedding ${\mathrm{emb}}_{t}$, defined as follows:

(7) $$\mathrm{PE}\left(t,i\right)=\left\{\begin{array}{cc} \sin(t\cdot w_{k}), & \mathrm{if}\;i=2k,\\ \cos(t\cdot w_{k}), & \mathrm{if}\;i=2k+1; \end{array}\right.$$

where $w_{k}$ = ${1/10,000}^{2k/d}$ and *d* is the encoding dimension. The class embedding ${\mathrm{emb}}_{c}$ is projected through a linear layer to match the hidden feature dimension of the denoising network, and together with ${\mathrm{emb}}_{t}$, serves as the conditioning input for noise prediction. In the reverse denoising process, the network predicts the noise component under both temporal and class conditions:

(8) $$\hat{\epsilon }=\epsilon_{\theta }\left(x_{t},t,c\right)$$

where $\epsilon_{\theta }\left(\cdot \right)$ denotes the parameterized denoising network. We implement it as an encoder-decoder U-Net. The encoder, built with stacked ResNet blocks and linear attention modules, progressively downsamples the input to enlarge the receptive field and capture temporal dependencies. The decoder further upsamples the latent representation and fuses it with multi-scale encoder features through skip connections under the guidance of conditioning embeddings to produce the final noise prediction $\hat{\epsilon }$.

#### 3.2.3. Temporal Data Generation with Classifier-Free Guidance

During inference, we employ classifier-free guidance to generate multimodal temporal segments conditioned on a specified motion class. The reverse process is initialized from Gaussian noise $x_{T}∼N\left(0,I\right)$ and proceeds iteratively from t=T to t=1. At each diffusion step, the denoising network produces both a conditional noise prediction ${\hat{\epsilon }}_{\theta }\left(x_{t},t,c\right)$ and an unconditional prediction ${\hat{\epsilon }}_{\theta }\left(x_{t},t\right)$. The difference between these predictions is used to construct the guidance term:

(9) $$\begin{array}{cc} \Delta \hat{\epsilon } & ={\hat{\epsilon }}_{\theta }\left(x_{t},t,c\right)-{\hat{\epsilon }}_{\theta }\left(x_{t},t\right) \end{array}$$

The final guided noise estimate is given by the following:

(10) $$\begin{array}{cc} {\hat{\epsilon }}_{t} & ={\hat{\epsilon }}_{\theta }\left(x_{t},t\right)+s\cdot \Delta \hat{\epsilon } \end{array}$$

where *s* denotes the guidance scale. Based on this guided noise estimate ${\hat{\epsilon }}_{t}$, an estimate of the clean sample ${\hat{x}}_{0}$ can be obtained as follows:

(11) $${\hat{x}}_{0}=\frac{x_{t}-\sqrt{1-{\bar{\alpha }}_{t}}\;{\hat{\epsilon }}_{t}}{\sqrt{{\bar{\alpha }}_{t}}}$$

where ${\bar{\alpha }}_{t}$ denotes the cumulative diffusion coefficient at step *t*. To improve sampling stability and prevent extreme values, ${\hat{x}}_{0}$ is clipped to the interval [−1,1]. Using the clipped estimate ${\hat{x}}_{0}$, together with the current noisy sample $x_{t}$ and the diffusion coefficients at step *t*, we compute the mean and variance of the reverse posterior distribution $q(x_{t-1}|x_{t},{\hat{x}}_{0})$. The posterior mean $\mu_{t}$ is expressed as follows:

(12) $$\mu_{t}=\frac{\sqrt{{\bar{\alpha }}_{t-1}}\;\beta_{t}}{1-{\bar{\alpha }}_{t}}\cdot {\hat{x}}_{0}+\frac{\sqrt{\alpha_{t}}\left(1-{\bar{\alpha }}_{t-1}\right)}{1-{\bar{\alpha }}_{t}}\cdot x_{t}$$

where $\beta_{t}=1-\alpha_{t}$ is the variance schedule coefficient. The corresponding posterior variance $\sigma_{t}^{2}$ is given by the following:

(13) $$\sigma_{t}^{2}=\frac{1-{\bar{\alpha }}_{t-1}}{1-{\bar{\alpha }}_{t}}\;\beta_{t}$$

The sample z∼N(0,I) at the previous step is then obtained by the following:

(14) $$x_{t-1}=\mu_{t}+\sigma_{t}\cdot z$$

At the final reverse step, we set *z* = **0** and directly use the posterior mean as the generated sample $x_{0}$.

To exploit the generated data for training, the synthetic temporal segments are combined with real samples to construct an augmented dataset. Specifically, for each motion class *c*, synthetic segments containing both the sensor sequence and the corresponding joint-moment target are generated according to a predefined augmentation ratio ρ and merged with the corresponding real segments. This class-wise augmentation strategy increases data diversity and improves category balance while preserving the underlying structure of the real multimodal temporal distribution.

### 3.3. Transformer-Based Joint Moment Estimation

Given the real and synthesized multimodal temporal segments, we employ a Transformer encoder-based regression network for end-to-end joint moment estimation. By leveraging multi-head self-attention, the network can model long-range temporal dependencies across the input window and capture interactions among heterogeneous sensor modalities, enabling joint prediction of hip and knee moments from multimodal time-series data.

Given a temporal segment, either a real sample *x* or a synthetic sample $x^{\prime }$, we partition it along the feature dimension into a multimodal sensor sequence $x^{s}\in R^{T_{s}\times D_{s}}$ and the corresponding joint-moment sequence $x^{m}\in R^{T_{s}\times 2}$. We extract a cropped sub-segment ${\tilde{x}}^{s}\in R^{T_{c}\times D_{s}}$ as the input to the regression network and obtain the aligned ground-truth joint-moment sequence ${\left\{m_{i}^{*}\right\}}_{i=1}^{T_{c}}\subseteq R^{2}$ from $x^{m}$, where $T_{c}<T_{s}$ denotes the effective input length. The cropped sensor sequence is then linearly projected into a latent feature space of dimension *C*, yielding the temporal representation $h\in R^{T_{c}\times C}$. To preserve temporal order information, a sinusoidal positional encoding ${\mathrm{emb}}_{pos}$, defined in Equation (7), is added element-wise to the projected features, resulting in the initial encoder input:

(15) $$z^{\left(0\right)}=h+{\mathrm{emb}}_{pos}$$

The sequence representation $z^{\left(0\right)}$ is subsequently fed into a Transformer encoder composed of *L* stacked layers for temporal feature extraction and refinement. In each layer, a multi-head attention (MHA) module is applied to model dependencies among all time steps, followed by a residual connection and layer normalization:

(16) $$\begin{array}{cc} {\tilde{z}}^{\left(l\right)} & =\mathrm{LN}(z^{\left(l-1\right)}+\mathrm{MHA}\left(z^{\left(l-1\right)}\right)) \end{array}$$

where LN(·) denotes the layer normalization operation. The resulting features are then processed by a feedforward network (FFN):

(17) $$\begin{array}{cc} z^{\left(l\right)} & =\mathrm{LN}({\tilde{z}}^{\left(l\right)}+\mathrm{FFN}\left({\tilde{z}}^{\left(l\right)}\right)) \end{array}$$

After passing through *L* encoder layers, the final high-level representation $z^{\left(L\right)}\in R^{T_{c}\times C}$ is fed into a linear regression head to generate the predicted moment sequence ${\left\{m_{i}\right\}}_{i=1}^{T_{c}}\subseteq R^{2}$, where the two output dimensions correspond to the hip and knee joint moments, respectively.

### 3.4. Loss Function

The proposed framework is trained in two stages. First, the classifier-free conditional diffusion model is optimized to learn class-conditioned multimodal temporal distributions and generate synthetic samples for data augmentation. The Transformer-based joint moment estimation network is then trained on the augmented dataset for end-to-end regression of hip and knee joint moments.

For the conditional diffusion model, we minimize the mean-squared error between the predicted noise and the ground-truth Gaussian noise:

(18) $$L_{\mathrm{diff}}=E_{x_{0},t,\epsilon }\left[{∥\epsilon -\epsilon_{\theta }\left(x_{t},t,c\right)∥}_{2}^{2}\right]$$

where ϵ denotes the Gaussian noise injected during the forward diffusion process, and $\epsilon_{\theta }\left(x_{t},t,c\right)$ is the predicted noise at diffusion step *t* under class condition *c*. By minimizing this objective, the model learns the conditional denoising mapping across diffusion steps and thereby approximates the underlying class-conditioned temporal data distribution.

For the joint moment estimation network, the regression objective is defined as the mean squared error over the temporal segment:

(19) $$L_{\mathrm{mom}}=\frac{1}{T_{c}}\sum_{i=1}^{T_{c}}{∥m_{i}-m_{i}^{*}∥}_{2}^{2}$$

where $m_{i}$ and $m_{i}^{*}$ denote the predicted and ground-truth joint moments, respectively. Minimizing this loss encourages accurate frame-wise estimation of both hip and knee joint moments over the entire temporal window.

## 4. Experiments

### 4.1. Experimental Settings

#### 4.1.1. Dataset

We evaluate the proposed framework on a publicly available multimodal lower-limb biomechanics and wearable sensor dataset [30]. The dataset contains time-series recordings from 22 healthy subjects performing a diverse set of lower-limb activities, covering 28 activity categories and 66 experimental conditions that can be broadly grouped into cyclic, impedance-like, and unstructured motions. During each trial, synchronized multimodal sensor signals, including IMUs, motor encoders, and plantar-pressure insoles, are recorded as subjects performed various lower-limb activities. The corresponding ground-truth joint moments are computed in OpenSim from motion-capture measurements and ground reaction force data. Following the official dataset split, we use the training set for both diffusion-model learning and joint moment estimator training, and reserve the test set exclusively for evaluation. The training set contains 1669 variable-length sequences from 15 subjects, whereas the test set contains 752 sequences from 10 subjects. Three subjects appear in both the training and test sets, but their data were collected under different exoskeleton assistance conditions. Moreover, no experimental conditions overlap between the training and test sets for the same subject. Therefore, the evaluation reflects generalization across unseen subject-condition combinations under the official dataset split, although it is not strictly subject-exclusive. In our experiments, we use IMU signals from the thigh, shank, and foot, encoder readings from the hip and knee joints, and plantar-pressure insole signals as the model inputs, resulting in a total of 25 sensor dimensions.

#### 4.1.2. Implementation Details

For the conditional diffusion module, each input segment has length $T_{s}=512$ and total feature dimension D=28, including $D_{s}=25$ dimensions for multimodal sensor signals, 2 dimensions for the joint moments, and 1 dimension for body weight. In classifier-free conditional modeling, the gating variable *g* is sampled with probability 0.5. The denoising network is implemented as a U-Net with 4 levels in both the encoder and decoder, using a base channel dimension of 64. During generation, the classifier-free guidance scale *s* is set to 3, and synthetic samples are merged with real samples using an augmentation ratio ρ=2. The activity-category distribution before and after diffusion-based augmentation is reported in Table 1. For the Transformer-based regression module, all input sequences are normalized using the mean and standard deviation computed only from the real training samples, and the same statistics are applied to the test set during evaluation. For the vertical plantar-force channel measured by the plantar-pressure insole, its values are first divided by the corresponding body weight before normalization. The cropped input sequence length $T_{c}$ is set to 200 with the latent feature dimension C=128. The Transformer encoder consists of 3 stacked layers with 4 attention heads.

The proposed Diff-MomentFormer framework is implemented in PyTorch 2.9.1 and trained on two NVIDIA GTX 3090 GPUs. For the diffusion model, the number of diffusion steps *T* is set to 1000. The model parameters are optimized using Adam with a batch size of 512 for 1,000,000 iterations, and the learning rate is fixed at $1\times 10^{-4}$ throughout training. For the joint moment estimation network, Adam is also adopted with a weight decay of $1\times 10^{-5}$ and a batch size of 128 for 500 epochs in total. The initial learning rate is set to $1\times 10^{-4}$, and an adaptive decay strategy with patience 10 and decay factor 0.9 is used. Early stopping with patience 50 is applied to reduce overfitting, and the Transformer dropout rate is set to 0.1. The resulting joint moment estimation network can run at a rate of 16,366.6 FPS with a single GPU during inference.

To comprehensively evaluate the effectiveness of the overall framework and its individual components, we compare the proposed method with a temporal convolutional network (TCN) as described in [29,30], which serves as a simple yet effective baseline for end-to-end joint moment estimation. Because the original implementation code in [30] is not publicly available, we re-implement the TCN model and train it under the same settings as the proposed regression module to ensure a fair comparison. Note that the primary focus of this work is to investigate the effectiveness of controllable data generation and model architecture. Therefore, we do not incorporate other performance-enhancing techniques, as they are considered orthogonal to the objectives of this study. For quantitative evaluation, we report several metrics including root mean square error (RMSE), coefficient of determination ($R^{2}$), and normalized mean absolute error (nMAE).

### 4.2. Quantitative Results

We first compare Diff-MomentFormer with the baseline TCN and two intermediate variants, namely MomentFormer, which improves only the regression backbone, and Diff-TCN, which only introduces diffusion-based data augmentation. The overall quantitative results are shown in Table 2. Diff-MomentFormer achieves the best performance across all evaluation metrics for both hip and knee joint moment estimation, demonstrating the effectiveness of integrating controllable data augmentation with Transformer-based temporal modeling. In particular, compared with TCN, Diff-MomentFormer reduces hip RMSE from 0.1810 to 0.1587 and knee RMSE from 0.1476 to 0.1261. MomentFormer already improves over the baseline, especially for knee joint estimation. This can be attributed to the superior ability of the Transformer encoder in modeling long-range dependencies and cross-modal correlations compared with the convolutional temporal backbone. Diff-TCN also consistently outperforms TCN, indicating that diffusion-based data augmentation enriches the training distribution and improves generalization. Notably, Diff-MomentFormer further surpasses both intermediate variants on all metrics, suggesting that the benefits of architectural improvement and synthetic data augmentation are complementary.

To further assess model behavior under different motion conditions, we compare the methods across activity categories using RMSE, as shown in Table 3. For each model, we repeated training five times with different random seeds and report the mean RMSE together with the standard deviation. Diff-MomentFormer achieves the lowest error in all three categories, including cyclic, impedance-like, and unstructured motions. For cyclic activities, the proposed method reduces hip RMSE from 0.1550 to 0.1380 and knee RMSE from 0.1471 to 0.1133 relative to the TCN baseline. More importantly, under impedance-like and unstructured activities, which involve greater variability, stronger environmental interaction, and more frequent motion transitions, Diff-MomentFormer continues to outperform all competing methods for both hip and knee joint moment estimation. The relatively small standard deviations across repeated runs further indicate that these performance trends are stable and consistent, even under the more challenging activity conditions. These results indicate that the proposed framework remains effective in more challenging and less regular scenarios, where the combination of richer synthetic training data and stronger temporal modeling helps capture complex multimodal dynamics and improves robustness across activity types.

Additionally, to assess whether the generated data preserve the statistical characteristics of real motions, we selected Walk (cyclic), Sit-to-Stand (impedance-like), and Start-and-Stop (unstructured), and compared the mean and standard deviation of two representative features for each activity, as shown in Table 4. Overall, the synthetic data remain close to the real data, indicating that the proposed model can generate physiologically reasonable signal magnitudes across different motion conditions. The discrepancy is smallest for Walk, larger for Sit-to-Stand, and largest for Start-and-Stop. We attribute this trend to differences in motion variability: walking is highly repetitive and consistent across sequences, whereas Start-and-Stop is much more diverse. The reduced variation in the synthetic data therefore appears to reflect a compression of this high variability while preserving the dominant statistical patterns of the activity.

### 4.3. Qualitative Results

**Figure 3:**
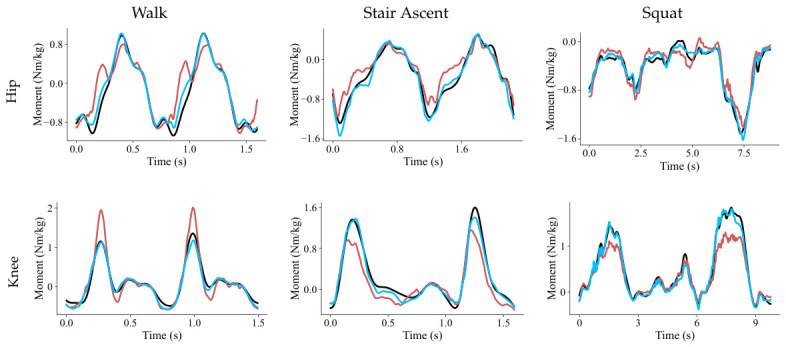
Qualitative comparison of hip and knee joint moment trajectories predicted by TCN and Diff-MomentFormer across multiple movement tasks. The red, blue, and black curves denote the predictions of TCN, the predictions of Diff-MomentFormer, and the ground-truth joint moments, respectively.

To qualitatively evaluate the proposed framework, we visualize the hip and knee joint moment trajectories predicted by TCN and Diff-MomentFormer, as shown in Figure 3. For cyclic movements such as walking and stair ascent, Diff-MomentFormer aligns with the ground-truth trajectories more closely in terms of waveform shape, amplitude variation, and temporal synchronization, indicating improved modeling of repetitive motion patterns. For the more challenging squat task, which exhibits stronger nonlinearity and larger local variations, Diff-MomentFormer still provides more accurate and stable predictions, especially during transition phases where the joint moment changes rapidly. In contrast, TCN shows larger deviations from the ground truth in both amplitude and timing. These observations are consistent with the quantitative results and further demonstrate the advantage of Diff-MomentFormer in robust multi-joint moment estimation across diverse movement conditions.

**Figure 4:**
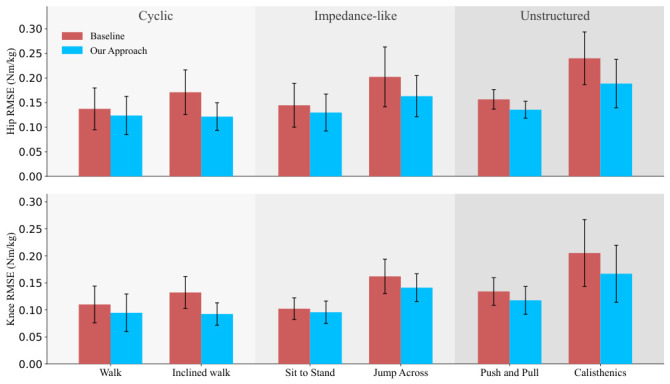
RMSE of the estimated hip (**top**) and knee (**bottom**) joint moments for selected activities, comparing the baseline method and the proposed approach. Bars denote the mean RMSE for each activity, and error bars indicate the corresponding standard deviation across different sequences.

In addition to the representative waveform comparisons in Figure 3, Figure 4 provides a category-wise visualization of the prediction error for selected activities from the cyclic, impedance-like, and unstructured motion groups. For both hip and knee joint moment estimation, the proposed approach consistently achieves lower RMSE than the baseline across all selected activities. The error bars further show that the proposed method generally maintains comparable or smaller variability across different sequences within the same activity, indicating improved robustness and generalization under diverse motion conditions.

**Figure 5:**
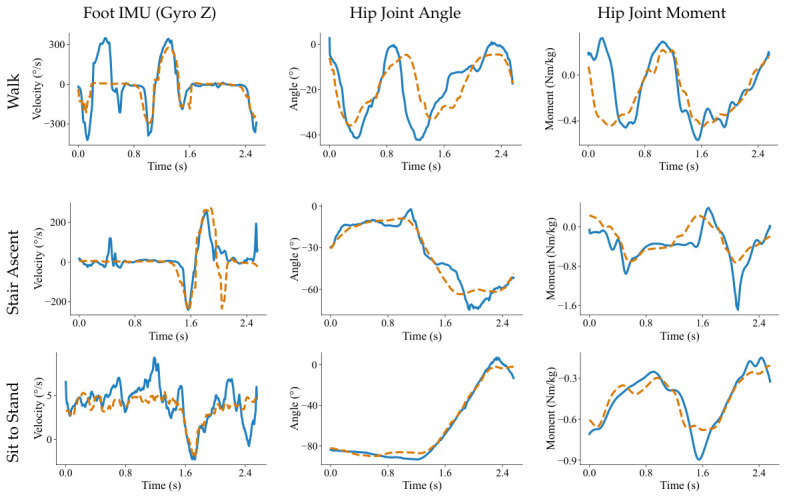
Qualitative comparison of synthetic and real multimodal time-series data across representative movement tasks. The blue solid and orange dashed lines represent the synthetic and real data, respectively.

To better understand the source of these performance gains, we further visualize representative synthetic data generated under several movement modes, as shown in Figure 5. Specifically, selected dimensions from multimodal signals, including the foot IMU gyroscope signal, hip joint angle, and hip joint moment, are compared with visually similar real samples from the same motion category. Benefiting from class-conditioned diffusion modeling, the generated signals capture the underlying motion regularities and cross-modal dependencies in the real data, rather than merely reproducing similar waveform shapes. For cyclic tasks such as walking and stair ascent, the generated signals preserve the overall temporal patterns and remain comparable to the real data in amplitude. Moreover, for a more complex movement such as sit-to-stand, the proposed method still captures the major motion transitions, suggesting its ability to model less regular and more transitional dynamics. These observations indicate that the proposed conditional diffusion model preserves physically meaningful multimodal temporal structure across subjects and movement states, thereby providing informative synthetic samples for downstream joint moment estimation.

### 4.4. Ablation Study

To further investigate the source of the performance gains, we conduct ablation studies on sensor configurations and synthetic data ratios. These experiments are designed to evaluate how different multimodal input settings and augmentation strategies affect joint moment estimation performance.

#### 4.4.1. Sensor Configurations

To examine the contribution of different sensor modalities to joint moment estimation, we conduct an ablation study on sensor configurations; the results are shown in Table 5. For each setting, we compute the RMSE values of hip and knee joint moments for each sequence and report the average results over the test set. (a), (b), and (c) use only a single sensor modality. Under the single-modality setting, using only IMU or encoder data yields substantially better performance than using only insole data, indicating that plantar pressure information alone is insufficient for accurate joint moment estimation. At the same time, IMU and encoder signals exhibit comparable effectiveness when used individually, although IMU data perform slightly better for hip moment estimation, whereas encoder inputs are marginally more beneficial for knee moment estimation. In (d), (e), and (f), combining two sensor modalities consistently outperforms the corresponding single-modality settings, demonstrating the benefit of multimodal fusion. In particular, the combination of IMU and encoder signals yields the best performance among the two-modality settings, suggesting that these two sources provide the most informative complementary cues for lower-limb joint moment estimation. The best overall performance is achieved when all sensor modalities are used together, which further confirms the complementary roles of different sensor types and the importance of multimodal integration in the proposed framework.

#### 4.4.2. Synthetic Data Ratios

**Figure 6:**
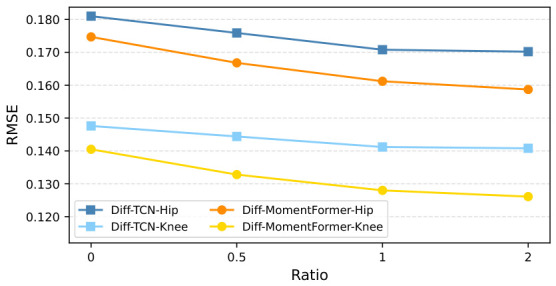
Hip and knee RMSE values of Diff-TCN and Diff-MomentFormer under different synthetic-to-real data ratios.

We further analyze the effect of different synthetic-to-real data ratios on model performance, as shown in Figure 6. As the proportion of synthetic data increases, both Diff-TCN and Diff-MomentFormer achieve lower RMSE values for hip and knee joint moment estimation, indicating that the diffusion model generates informative samples that effectively enrich the training distribution. However, the improvement of Diff-TCN becomes limited as the ratio increases and is nearly saturated when the ratio reaches 1, suggesting that the TCN-based backbone cannot continue to benefit substantially from additional synthetic data beyond this point. In contrast, Diff-MomentFormer shows more consistent performance gains as the synthetic data ratio increases and continues to improve even at higher ratios. Consequently, the performance gap between Diff-MomentFormer and Diff-TCN becomes more pronounced with increasing amounts of synthetic data. These results demonstrate that the Transformer-based backbone is better able to exploit the diversity introduced by diffusion-based augmentation and benefits more consistently from increasing amounts of synthetic data. Overall, the ablation results further confirm that both class-conditioned synthetic data generation and Transformer-based temporal modeling are critical to the final performance gains of Diff-MomentFormer.

## 5. Conclusions

In this paper, we propose Diff-MomentFormer, a generative diffusion-augmented Transformer framework for end-to-end joint moment estimation. The proposed method integrates a classifier-free conditional diffusion model for class-conditioned multimodal temporal data generation with a Transformer-based regression network for long-range temporal modeling and cross-modal feature fusion. Extensive experiments on a public lower-limb biomechanics dataset demonstrate that Diff-MomentFormer consistently improves hip and knee joint moment estimation performance across different activity categories and sensor configurations. These results confirm that the combination of class-conditioned synthetic data augmentation and Transformer-based temporal modeling is effective for improving both the accuracy and robustness of end-to-end joint moment estimation. Overall, this work provides valuable insights into multimodal biomechanical sequence modeling and may support future work on integrating this approach into data-driven lower-limb exoskeleton control systems, where joint moment estimation is a key component.

## Figures and Tables

**Table 1:** Activity-category distribution of samples before and after diffusion-based augmentation. The main values indicate the number of samples, while values in parentheses indicate the percentage of each activity category. The imbalance ratio is defined as the largest-to-smallest activity-category count ratio.

Dataset	Cyclic	Impedance-Like	Unstructured	Imbalance Ratio
Before	1,729,000 (69.4%)	420,000 (16.8%)	344,000 (13.8%)	5.03:1
After	3,391,000 (45.3%)	2,082,000 (27.8%)	2,006,000 (26.8%)	1.69:1

**Table 2:** Comparison of different methods for hip and knee joint moment estimation on the test set in terms of RMSE, $R^{2}$ and nMAE. ↓ indicates that lower values are better, while ↑ indicates that higher values are better. The best results are in bold.

Method	Hip			Knee		
	RMSE ↓	$R^{2}$ ↑	nMAE ↓	RMSE ↓	$R^{2}$ ↑	nMAE ↓
TCN	0.1810	0.6319	9.33	0.1476	0.7340	7.01
MomentFormer	0.1747	0.6360	9.21	0.1405	0.7609	6.43
Diff-TCN	0.1702	0.6485	8.87	0.1408	0.7486	6.68
Diff-MomentFormer	**0.1587**	**0.6952**	**8.25**	**0.1261**	**0.7893**	**6.06**

**Table 3:** RMSE comparison of different methods under different activity categories. For each method, the first line reports the mean RMSE over five runs with different random seeds, and the second line in parentheses reports the corresponding standard deviation. The best results are in bold.

Method	Cyclic		Impedance-Like		Unstructured	
	Hip	Knee	Hip	Knee	Hip	Knee
TCN	0.1550	0.1471	0.1862	0.1416	0.2118	0.1608
	(0.0013)	(0.0010)	(0.0017)	(0.0011)	(0.0027)	(0.0015)
MomentFormer	0.1532	0.1220	0.1753	0.1345	0.2046	0.1501
	(0.0008)	(0.0005)	(0.0016)	(0.0009)	(0.0025)	(0.0013)
Diff-TCN	0.1492	0.1412	0.1798	0.1364	0.1991	0.1525
	(0.0011)	(0.0008)	(0.0015)	(0.0011)	(0.0025)	(0.0015)
Diff-MomentFormer	**0.1380**	**0.1133**	**0.1582**	**0.1256**	**0.1892**	**0.1461**
	(0.0007)	(0.0004)	(0.0012)	(0.0009)	(0.0020)	(0.0010)

**Table 4:** Statistical comparison between real and synthetic data for representative features under different motion categories. One representative activity was selected from each category, namely Walk (cyclic), Sit-to-Stand (impedance-like), and Start-and-Stop (unstructured). For each activity, two representative features are reported in terms of mean and standard deviation.

Class	Feature	Mean ± Std	
		Real	Synthetic
Walk	shank_imu_gyro_x	−7.73 ± 22.53	−7.33 ± 18.38
	insole_force_y	550.55 ± 454.54	526.82 ± 432.79
Sit-to-Stand	foot_imu_gyro_z	−5.48 ± 8.37	−5.95 ± 5.34
	shank_imu_accel_z	−1.28 ± 0.86	−1.28 ± 0.49
Start-and-Stop	foot_imu_gyro_z	−4.00 ± 131.52	−4.77 ± 50.01
	foot_imu_accel_y	10.39 ± 4.56	9.72 ± 1.96

**Table 5:** Ablation study of different sensor configurations for joint moment estimation. ✓ indicates that the corresponding sensor modality was used as input. The best results are in bold.

	Insole	Encoder	IMU	RMSE	
				Hip	Knee
(a)	✓			0.2896	0.2615
(b)		✓		0.1605	0.1319
(c)			✓	0.1529	0.1328
(d)	✓	✓		0.1560	0.1266
(e)	✓		✓	0.1524	0.1292
(f)		✓	✓	0.1428	0.1189
(g)	✓	✓	✓	**0.1383**	**0.1135**

## Data Availability

The dataset [30] used in this article is publicly accessible.

## Abbreviations

The following abbreviations are used in this manuscript:

IMU	Inertial Measurement Unit
GRF	Ground Reaction Force
TCN	Temporal Convolutional Network
CNN	Convolutional Neural Network
ViT	Vision Transformer
DETR	Detection Transformer
DiT	Diffusion Transformer
LDM	Latent Diffusion Models
DDIM	Denoising Diffusion Implicit Model
MHA	Multi-Head Attention
FFN	Feedforward Network
RMSE	Root Mean Square Error
nMAE	Normalized Mean Absolute Error
